# Legal rights of client councils and their role in policy of long-term care organisations in the Netherlands

**DOI:** 10.1186/1472-6963-11-215

**Published:** 2011-09-12

**Authors:** Marloes Zuidgeest, Katrien G Luijkx, Gert P Westert, Diana MJ Delnoij

**Affiliations:** 1 Tranzo, Academic Research Centre for Health and Social Care, Faculty of Social and Behavioural Sciences, Tilburg University, PO Box 90153, 5000 LE Tilburg, The Netherlands; 2 IQ Healthcare, Scientific Institute for Quality of Healthcare, Radboud University Nijmegen Medical Centre, PO Box 9101, 114, 6500 HB Nijmegen, the Netherlands; 3 Centre for Consumer Experience in Health Care, P.O. Box 1568, 3500 BN, Utrecht, The Netherlands

**Keywords:** Consumer participation, empowerment, patients' rights, long-term care

## Abstract

**Background:**

Legislation demands the establishment of client councils in Dutch nursing homes and residential care facilities. The members of those councils are residents or their representatives. Client councils have the right to participate in the strategic management of long-term care facilities. More specifically, they need to be consulted regarding organisational issues and a right to consent on issues regarding daily living of residents, including CQ-index research. CQ-index research concerns a method that measures, analyses and report clients' experiences about the quality of care. Research questions were: '*Do client councils exercise their rights to be consulted and to give their consent?' *and '*What is the role of client councils in the process of measuring clients' experiences with the CQ-index and what is their opinion about the CQ-index?'*

**Methods:**

Postal questionnaires were sent to members of 1,540 client councils of Dutch nursing homes and residential care facilities. The questionnaire focussed on background information and client councils' involvement in decision-making and strategic management.

**Results:**

The response rate was 34% (n = 524). Most councils consisted of seven members (range: 5 to 12 members). One out of four members participating in the client councils were clients themselves. Although councils have a legal right to be consulted for organisational issues like finance, vision, annual report, and accommodation, less than half the councils (31-46%) reported that they exercised this right. The legal right to consent was perceived by 18 to 36% of the councils regarding client care issues like food and drink, complaints registration, respectful treatment, and activities. For CQ-index research, only 18% of the client councils perceived a right to consent. Their rights to choose an approved contractor -who performs CQ-index research- and indicating improvement priorities, were hardly used.

**Conclusions:**

Client councils play a rather passive role in determining the policy on quality of long-term care. Therefore, specific attention and actions are needed to create a more proactive attitude in councils towards exercising their rights, which are already supported by legislation.

## Background

In the Dutch healthcare system, the emphasis is shifting from provider domination to client orientation [1,2], where the role and position of clients have been strengthened in recent decades in a number of ways including legislation [3]. For example, the Dutch 'Participation by clients of Care Institutions Act ('Wet Medezeggenschap Cliënten Zorginstellingen; WMCZ)' mandates *every *healthcare organization to have a functioning client advisory council; a board whose members are recruited from the users of the organization and who will represent them [4]. In practice, most councils have between five and ten members, depending on the size of the organization. In nursing homes and residential care facilities, spouses (of deceased clients) and volunteers are members of these councils along with clients themselves. Healthcare organisations facilitate these councils by providing resources such as office space and equipment, meeting rooms, budget, et cetera. [5]. The WMCZ gives client councils the right to advise the management of the organisation about quality of care, and the law prescribes that the healthcare organisation asks for this advice.

More specifically, according to this law councils have been granted the following rights: to have meetings with management about organisation policy, to receive information, to request an investigation into mismanagement, to be consulted, and the right to consent [4]. The right to be consulted allows councils to give their advice regarding issues on changing the aim and policy of the organisation, merger with another organisation, and financial matters, but the management can ignore this advice. The right to consent implies that councils need to formally approve plans concerning issues that affect the daily living of clients (food and drink, safety, recreation and leisure), hygiene, the quality of healthcare for residents, changes to the complaints procedure, and Consumer Quality Index (CQ-index or CQI) research. The management cannot carry out changes regarding these issues without approval of the client council [4,6]. CQ-index is a standard methodology used in the Netherlands to measure, analyse, and report experiences of clients regarding the quality of healthcare. Besides this, the methodology includes also protocols for developing new CQI questionnaires. Questionnaires can be developed to assess the quality of care of a sector, professionals or treatment of a disease [7,8].

The CQ-index for the nursing and caring sector -the CQI 'Long-term Care'- [9] consist of several questionnaires targeting various client populations and domestic settings with tailored data collection: a face-to-face interview protocol for residents (1), a mail questionnaire for representatives of psychogeriatric clients (2), and a mail questionnaire for assisted-living clients (3). Outcomes of these questionnaires (also called client-related indicators) together with clinical indicators (e.g. incidence of skin ulcers, falls, malnutrition, and medication errors) form the national Quality Framework of Responsible Care [10]. This information is also disclosed on the Internet, which creates transparent information about providers' performance [11]. The framework consists of 19 indicators on four domains: a) quality of life, b) quality of caregivers, c) quality of care organization, and d) technical aspects. The CQ-index relates to indicators in the first three domains. Biennially, CQI data are collected, analysed, and reported by approved contractors. These contractors meet certain minimum performance standards based on ISO 20252, which is an international standard for market, opinion and social research. Healthcare organisations can choose any approved contractor. These contractors differ in price and the type of information products they provide. Some offer feedback reports that only report on the findings for one's own organisations, others offer benchmark reports in which one's own performance is compared to that of other organisations. Some contractors offer the opportunity to have an additional presentation of the findings, for instance, for the client council and/or for staff members. Because CQI research provides quality information regarding residents' daily living, councils have the right to consent to choose an approved contractor, but they have also a voice in pointing out improvement activities based on CQI results.

In 2004, an evaluation of the effect of client councils on decision-making of the organisation showed that councils influence on issues covered by the right to consent was small. According to a representative group of client councils in nursing homes, only half of these councils were given notice of decisions on which they had right to consent [12].

Research about the role of client councils in the Netherlands is scarce and we do not know whether and to what extent client councils use their rights. Therefore, our first research question is: '*Do client councils exercise their rights to be consulted and to give their consent?' *Because the CQ-index is relatively new, we were particularly interested in the role of client councils in the process of measuring client experiences and their opinion about the CQ-index. Therefore, our second research question is '*What is the role of client councils in the process of measuring clients' experiences with the CQ-index and what is their opinion about the CQ-index?*

## Methods

In 2010, we sent 1,540 postal questionnaires to contact persons of client councils in nursing homes and residential care facilities about the use and usability of CQI information. Addresses were obtained via the Nationwide organisation for client councils (LOC Zeggenschap in Zorg). All client councils in nursing homes and homes for the elderly are members of this organisation. However, only members who had given LOC permission to sent questionnaires were contacted. One reminder was sent as a 'thank you' card ten weeks after the initial postal questionnaire. The contact persons were informed about the aim of the questionnaire and were free to respond or not. According to the Dutch Medical Research Involving Human Subjects Act (WMO), ethical approval of the survey research was not necessary.

The questionnaires contained 1) background questions, 2) questions about councils' influence on organisational policy with 5 response categories 'totally not involved', 'only informative (client council receives only information which informs them, no action of council is required)', 'right to be consulted (client council has the right to give their advice, but the management can ignore this)', 'right to consent (client council needs to formally approve changes; this approval is mandatory and cannot be ignored by the management)', and 'Don't know', and 3) statements about the CQ-index with response categories on a 5-point Likert scale (ranging from 'totally disagree' to 'totally agree'). Descriptive analyses were performed using SPSS version 17.0 ^®^.

## Results

### Background information of client councils

The response rate to the postal questionnaire was 34% (n = 524). No information was available of contact persons who did not respond. Most responding councils consisted of seven members, with a minimum of five and maximum of twelve members. One out of four members participating in the client councils were residents themselves. Volunteers (25%), Family of residents (22%), family members of deceased residents (15%), and assisted living clients (13%) also participated in the councils.

Client council meetings occurred once in four to twelve weeks and meetings with the management were held less often (Table 1). In the meetings, they discussed topics regarding organisational issues -financial matters (82% of the councils), vision of the health organization (71%), annual report (77%), new employees (51%), accommodation (81%), and laundry costs (82%)- and topics regarding client care - organization of care (52%), food and drinks (89%), handling complaints (83%), respectful treatment and privacy of residents (76%), choice for improvement projects (75%), variety of activities (69%), and CQI research (74%)-.

**Table 1:** Meetings of client councils with members only and of members with the management

*Once per:*	*N*	*4 weeks*	*6 weeks*	*8 weeks*	*12 weeks*	*Different frequency*
Client council members only	515	51%	30%	14%	2%	4%
With management	513	36%	25%	27%	5%	7%

The councils' role varied with respect to the frequency of giving written advice to the management of organisations: 0 advices per year (28% of the councils), 1 to 5 advices per year (58%), 6-10 advices (11%), and more than 10 advices per year (3%). Quality improvement priorities were formulated by the client council alone (16%), by the management alone (21%), and by the management and client councils together (51%).

### Client councils and their involvement in decision-making

Table 2 provides information regarding the degree of involvement of councils in decision-making on organisational issues. With respect to these issues, client councils have the *right to be consulted*. This means that the management has to ask for the advice of the client council, but the management is free to ignore this advice. As can be seen in the Table, 31% to 46% of the councils exercised this right with respect to issues from finance to accommodation. However, almost the same percentage of respondents believes that their involvement in these issues is of an 'only informative' nature (23% to 46%). So, they believe that the management shares information about these with them, but they are not aware of the fact that the management should ask for their advice. On the other hand, 12 to 27% of the client councils report that they have the right to consent on these issues. When the percentages of the 'rights to be consulted' and 'right of consent' are summated, for almost every organisational issue more than 50% councils exercise their legal right or are even more involved than would be necessary from a legal point of view.

**Table 2:** The degree of involvement of client councils in decision-making on various topics for which they have the right to be consulted

*Issues*		*Not* *involved*	*Only informative*	*Right* *to be* *consulted*	*Right to consent*	*Don't know*
	N	%	%	%	%	%
Financial matters (e.g. budget)	505	10	45	31	12	2
Vision of the organisation	497	5	37	36	19	2
Annual report	500	6	46	34	12	2
New employee	499	25	23	33	18	2
Accommodation	498	5	30	46	17	3
Laundry costs	497	6	27	38	27	2

Table 3 displays issues concerning the councils' *right to consent *in decision-making of the healthcare organisation on client care (e.g. food and drink, complaints registration, respectful treatment and activities). With respect to these issues, the client council has to formally approve any plans or change of policy that the management proposes. Only 18% to 36% of the client councils experienced that they actually had this right. More client councils reported that with respect to these issues they had the 'right to be consulted' (31% to 50%). Some of the client councils (16% to 35%) even perceived their involvement with these issues as 'only informative'.

**Table 3:** The degree of involvement of client councils in decision-making on various topics for which they have the right to consent

*ISSUES*		*Not* *involved*	*Only informa tive*	*Right* *to be* *consulted*	*Right to consent*	*Don't know*
	N	%	%	%	%	%
Organisation of care	498	21	26	31	19	3
Food and drink	510	7	16	42	36	1
Complaints procedure	497	5	27	37	27	3
Respectful treatment and privacy	501	5	24	41	27	3
Choice of improvement projects	505	4	21	50	22	3
Variety of activities	508	8	32	36	21	2
CQI research	466	5	35	37	18	3

For the CQI survey and for choosing improvement projects, only 18% and 22% of the councils perceived that they had the right to consent, respectively. These results indicate that councils' degree of involvement in decision-making in healthcare organisation is less than expected based on their legal rights.

### Role of client councils in the process of measuring clients' experiences

If client councils are involved in CQI research they need information from the management about when a next measurement is to take place. Most of the councils were given timely notice when a new CQI survey was to start (87%): the preferred notice period was two months and three-quarters of the client councils were adequately informed about the CQI survey.

To ensure that measurements of client experiences are embedded in the decision-making process of the organisation, client councils have the right to consent regarding the selection of an approved contractor to perform the CQI survey. This enables client councils, for example, to choose a contractor who offers information products, such as reports and presentations that are tailored to client councils' needs. Twenty-nine percent of the respondents replied that they were involved in this selection; implicating 71% of the councils had no role in choosing a contractor. Among those client councils that found it important to be involved in this process (69%), 41% were involved.

Statements about measuring clients' experiences with the CQI method showed that respondents were positive about the CQ-index. Two-thirds of the client councils agreed that the CQI questionnaire was a good questionnaire to measure clients' experiences with care and almost the same percentage agreed that CQ-index provided clear questions. Although the CQ-index provided recognizable results, the councils disagreed about the extent to which results were representative. Seventy-six percent of the respondents think that results point out improvement potential (Table 4).

**Table 4:** Statements measuring clients' experiences with the CQI method

		*(totally)* *disagree*	*disagree*	*neutral*	*agree*	*(totally)* *agree*
	N	%	%	%	%	%
Results show improvement potential	453	2	4	9	67	18
There are recognizable results	443	2	7	23	59	9
CQ-index is a good questionnaire for measuring clients' experiences of care	448	3	6	25	55	11
Questions in the survey are clear	445	2	11	20	58	9
Results show a representative image of the experiences of clients	438	4	21	28	40	7

## Discussion

The aim of this article was two-fold. First, we assessed whether client councils exercise their legal rights in decision-making of nursing and residential facilities. More specifically, we looked at their rights to be consulted and to give their consent on several issues. Second, we examined what the role of client councils is in the process of measuring clients' experiences with the Consumer Quality index (CQ-index or CQI) 'Long-term Care' and what their opinion about this CQ-index is.

The respondents from client councils of nursing homes and residential care facilities were drawn from available addresses of the Nationwide organisation for client councils. These councils agreed to receive incidental questionnaires. In 2009, there were 2082 nursing homes and residential facilities in the Netherlands [13]. With our sample we reached 73% of the homes. No information was available of the councils that were not reached or did not respond to the questionnaire. The low response rate of the questionnaire (34%) may have biased the results. It is likely that the responses are too positive because a subgroup did not respond. For the non-respondents, CQI research is probably less well known and more complex than for the respondents. Continuing this line of argument, councils may use their legal voice less often than was presented in the results.

The composition of the councils in this study was the same as in the evaluation report of the Participation by clients of Care Institutions Act in 2004 [5]. Nevertheless, this composition - only one in four members is a client - needs attention [14]. When entering a home, clients are elderly and have physical complaints that limit them in joining the client council. This is a concern for the next decades, because client councils have a legal voice on policy regarding healthcare facilities. If clients are not able to use this voice individually or through a client council others must do so for them.

The involvement of client councils in decision-making of healthcare organisations is embedded in legislation. However, less than half (31%-46%) of the client councils perceived that they could exercise their *right to be consulted *on organisational issues like finance, vision, annual report and accommodation. Even fewer councils (18%-36%) perceived that they could exercise their *right to consent *about issues concerning client care (e.g. food and drink, complaints registration, respectful treatment and activities). The fact that not many councils are involved in decision-making and policy of healthcare organisations is in contrast with a national agreement between stakeholders about client council rights [6].

Concerning CQI research, client councils have the right to consent regarding the selection of an approved contractor to perform the CQI survey. However, our results showed that client councils did mostly not choose approved contractors. Client councils have hardly any role in the process of measuring clients' experiences with CQI surveys: only one fifth of the client councils perceived that they could exercise their right to consent regarding CQI research.

The literature shows that recognizable results promote the use of client feedback [15]. Despite the fact that the CQI results were recognizable, some client councils were not involved in formulating priorities for quality improvement. Management needs to notify councils when a CQI survey is to take place and should encourage councils to be more actively involved in pointing out own improvement priorities. Actively involving client councils in the identification of priorities and quality activities reduces the amount of undesirable outcomes (e.g. the prevalence of pressure ulcers, restricted mobility and behavioural problems) [16].

## Conclusions

In conclusion, measuring clients' experiences should constitute a mean to strengthen the position and role of clients in nursing homes and residential care facilities. Nevertheless, councils hardly use their legal voice and they tend to have a passive role. Measuring clients' experiences is part of client-oriented policy, aimed at improving quality of care, but the current practice shows that the management of healthcare organisations dominates the process involved. This is a top-down approach and conflicts with the national policy aimed at client empowerment.

## Competing interests

The authors declare that they have no competing interests.

## Authors' contributions

MZ conducted the study, analysed and interpreted the data, and wrote a draft manuscript. KL, GW and DD were involved in the design of the study and revised the manuscript critically. All authors read and approved the final manuscript.

## Pre-publication history

The pre-publication history for this paper can be accessed here:

http://www.biomedcentral.com/1472-6963/11/215/prepub
